# Systematic Review: Efficacy, Safety Profile, and Cost-Effectiveness of Nirsevimab Versus Palivizumab for RSV Prevention in Children Under 24 Months

**DOI:** 10.3390/children13030331

**Published:** 2026-02-26

**Authors:** Andreea Văduva, Alexandru Dinulescu, Anca Cristina Drăgănescu, Sorin Claudiu Man, Doina Anca Pleșca

**Affiliations:** 1 Department of Pediatrics, Faculty of Medicine, “Carol Davila” University of Medicine and Pharmacy, 050474 Bucharest, Romania; andreea.enoiu@drd.umfcd.ro (A.V.); anca.draganescu@umfcd.ro (A.C.D.); doina.plesca@umfcd.ro (D.A.P.); 2 “Matei Bals” National Institute of Infectious Diseases, Dr. Calistrat Grozovici Street, No 1, Sector 2, 021105 Bucharest, Romania; 3 Emergency Hospital for Children “Grigore Alexandrescu”, 011743 Bucharest, Romania; 4 Mother and Child Department, University of Medicine and Pharmacy “Iuliu Hatieganu”, 400347 Cluj-Napoca, Romania; claudiu.man@umfcluj.ro

**Keywords:** respiratory syncytial virus (RSV), bronchiolitis, nirsevimab, palivizumab, immunoprophylaxis, vaccines, monoclonal antibody

## Abstract

**Highlights:**

**What are the main findings?**

- Nirsevimab demonstrates comparable or superior efficacy to palivizumab in preventing respiratory syncytial virus lower respiratory tract infections in children.
- Compared with palivizumab, nirsevimab has a more favorable administration profile, requiring only a single dose per RSV season, and has a similar safety profile.

**What are the implication of the main findings?**

- The use of nirsevimab may improve adherence to RSV prophylaxis programs by reducing the need for multiple injections during the RSV season.
- Nirsevimab represents a potentially cost-effective alternative for large-scale RSV prevention strategies in infants and young children.

**Abstract:**

**Background**: Respiratory syncytial virus (RSV) is a leading cause of lower respiratory tract infections (LRTI) in infants and young children, with significant morbidity, hospitalizations, and healthcare costs. Passive immunoprophylaxis has historically relied on palivizumab, while nirsevimab, a long-acting monoclonal antibody, has recently emerged as an alternative and has broader indications. **Methods:** We conducted a systematic review of randomized controlled trials, observational studies, real-world effectiveness analyses, and economic evaluations assessing the efficacy, safety, and cost-effectiveness of palivizumab and nirsevimab for RSV prevention in children under 24 months. **Results:** 41 studies were included in this review. Palivizumab demonstrated consistent efficacy in reducing RSV-related hospitalizations in high-risk infants, with relative risk reductions of approximately 45–55%. Nirsevimab showed higher relative efficacy, with reductions of 70–85% in RSV-associated lower respiratory tract infections and hospitalizations across randomized trials and real-world studies, including healthy term and late-preterm infants. Both monoclonal antibodies have favorable safety profiles, with adverse event rates comparable to the placebo. Economic analyses indicated that palivizumab is cost-effective primarily in narrowly defined high-risk populations, whereas nirsevimab may offer improved cost-effectiveness, particularly at lower acquisition prices and with seasonal administration strategies. **Conclusions:** Nirsevimab represents a promising advancement in RSV prevention, with broader protection, a simpler administration, and potential economic advantages compared to palivizumab.

## 1. Introduction

Respiratory syncytial virus (RSV) remains the most frequent cause of acute lower respiratory tract infections (LRTI) in infants and young children worldwide, contributing to a substantial burden of morbidity, mortality, and healthcare resource utilization [1,2]. In a systematic review published by Shi et al. (2017) they estimated that in 2015 RSV was responsible of 33 million episodes of acute LRTI in children under 5 years old, 3.2 million hospital admission and 59,600 deaths worldwide, even though it seemed there was a decrease in this incidence during the COVID-19 pandemic, there are reports of a resurgence in the post-pandemic period [3,4,5,6,7,8]. The infection with RSV is particularly severe in high-risk groups, including premature infants, immunocompromised children, and those with chronic lung disease (CLD) or congenital heart defects (CHD) [9,10,11,12].

Until recently, palivizumab, a humanized monoclonal antibody licensed by the United States Food and Drug Administration (FDA) in 1998, was the only passive immunoprophylaxis against RSV [13,14,15,16]. However, its use in clinical practice has been limited by several factors: the requirement for monthly intramuscular injections throughout the RSV season, substantial cost, and only moderate efficacy [17,18,19].

Nirsevimab is a long-acting human monoclonal antibody engineered with an extended half-life, providing season-long protection with a single intramuscular dose [20,21]. It was approved by the European Medicines Agency (EMA) in 2022 and by the FDA in 2023 [22,23]. The prolonged half-life of nirsevimab relative to palivizumab is achieved through targeted amino acid substitutions in the Fc region (M252Y/S254T/T256E, collectively termed the “YTE” mutations), which increase binding affinity to the neonatal Fc receptor (FcRn) and thereby reduce catabolic degradation of the antibody [24]. The efficacy and safety profile of nirsevimab represents a promising alternative to palivizumab for RSV prevention [25,26,27].

This systematic review aims to evaluate and compare the efficacy, safety profile, and cost-effectiveness of nirsevimab compared to palivizumab in preventing RSV disease in children. Direct head-to-head comparisons between palivizumab and nirsevimab are limited, the two monoclonal antibodies having been applied in distinct cohorts (palivizumab in narrowly defined high-risk infants, whereas nirsevimab as a universal prophylactic approach). This analysis prioritizes indirect evidence on relative effectiveness, safety, and cost-effectiveness within approved indications and at the health-system level.

## 2. Materials and Methods

This systematic review was conducted and documented in accordance with the Preferred Reporting Items for Systematic Reviews and Meta-Analyses (PRISMA).

### 2.1. Literature Search Strategy

A comprehensive literature search was performed in PubMed/MEDLINE, Embase, and Web of Science to identify studies assessing the efficacy, safety, and cost-effectiveness of palivizumab and nirsevimab for preventing RSV infection in infants and young children. The search covered publications from database inception to the most recent available date and was restricted to publications in English. Manual reviews of the reference lists provided in our review articles, clinical trials, and health technology assessments of included studies also created additional opportunities to identify relevant studies related to childhood RSV prevention.

### 2.2. Eligibility Criteria

Eligible studies included randomized controlled trials (RCTs), observational cohort studies, real-world effectiveness and safety analyses, and economic evaluations (cost-effectiveness, cost-utility, and budget impact analyses). Publications were restricted to those enrolling infants and young children during their first or second RSV season. We excluded conference abstracts that did not include full-text data, case reports, and studies that did not report on relevant clinical or economic outcomes.

### 2.3. Studies Selection According to Outcome Domain

#### 2.3.1. Efficacy

To reflect effectiveness in routine clinical conditions, pivotal trials were supplemented with a small number of representative real-world and observational studies. Because of the long history of the production of palivizumab, we chose to focus only on the pivotal studies and representative studies that answered the same question to reduce redundancy (for effectiveness in the area of real-world effectiveness, we focused on larger representative cohort studies that captured aggregated RSV hospitalization in preterm infants (<35 weeks of gestation)). When available, systematic or pooled analyses were considered only if they added substantial complementary evidence not covered by individual large cohorts. This approach allowed us to include a smaller number of studies that demonstrated the real-world effectiveness of palivizumab while maintaining a necessary and small evidence base. For nirsevimab, however, we included all major phase 2/3 trials and the largest methodologically rigorous studies that provided evidence for real-world effectiveness. All direct head-to-head comparisons were included in the analysis when possible, and the findings were published individually.

#### 2.3.2. Safety

The primary sources of safety data were pivotal randomized controlled trials and large observational studies/post-marketing trials, which reported adverse effects, serious adverse effects, hypersensitivity effects, and mortality. For both monoclonal antibodies, studies with comparative safety data or long-term follow-up were prioritized. To improve the integrity of the evidence base and avoid redundancy, we excluded smaller or redundant studies with similar safety profiles.

#### 2.3.3. Cost-Effectiveness Data

The economic data were sourced from published cost-effectiveness and cost-utility Analyses, Systematic Review of Economic Evaluations, and National/Regional Health Technology Assessments. Studies were selected to reflect a range of healthcare system perspectives and target populations.

### 2.4. Data Extraction and Synthesis

From each included study, detailed information was extracted regarding study design, population characteristics, interventions and comparator groups, as well as clinical outcomes, including RSV-related hospitalizations and adverse events. Economic outcomes were also collected, encompassing financial impacts such as costs per unit of benefit gained and cost-utility measures expressed as cost per quality-adjusted life year (QALY).

**Figure 1:**
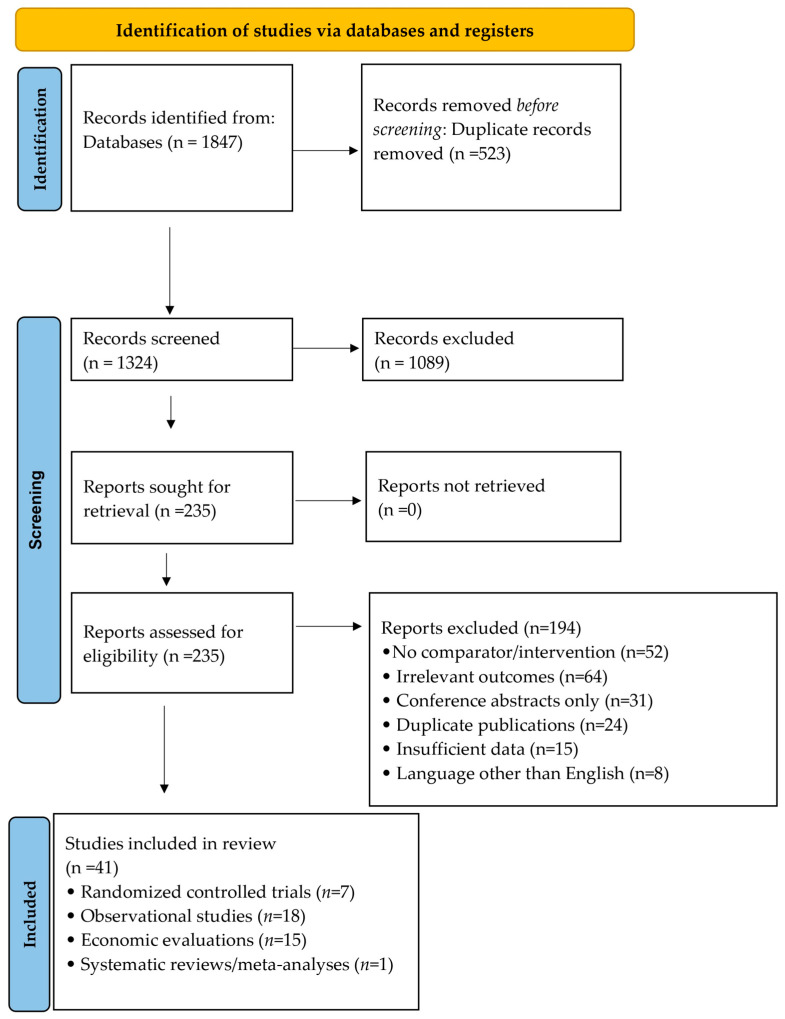
PRISMA Flow Chart.

The performance, hazards, and costs of palivizumab and nirsevimab were reported separately before being indirectly compared using descriptive analysis. There were 41 studies included in this review (Figure 1).

### 2.5. Risk of Bias Assessment

Judgments of potential flaws were based upon how studies were designed and executed. Trials that used random assignment typically showed minimal bias concerns; meanwhile, observation-based or real-life reports often carried moderate concerns because variables were not fully controlled. For cost–benefit models, the evaluation focused on clarity, including the explicit reporting of assumptions, the coherence of the analytical framework, and the use of sensitivity analyses to assess the robustness of the results.

## 3. Results

### 3.1. Clinical Efficacy

#### 3.1.1. Palivizumab Efficacy

The effectiveness of palivizumab was first established through the well-known IMpact-RSV study in 1998—a randomized, double-blinded, placebo-controlled trial that included 1502 infants at high risk for developing RSV. Palivizumab was shown to reduce RSV-related hospitalizations by 55% in premature babies <35 weeks gestational age and children with bronchopulmonary dysplasia (BPD) treated with monthly palivizumab injections, (10.6% in placebo group vs. 4.8% in palivizumab group, *p* < 0.001), resulting in a number needed to treat (NNT) of approximately 17 to prevent one RSV hospitalization [15]. In infants with hemodynamically significant congenital heart disease, the Feltes et al. (2003) randomized trial demonstrated a significant reduction in RSV-associated hospitalizations among those receiving palivizumab compared with placebo, confirming efficacy in this high-risk subgroup [28]. Data from the Palivizumab Outcomes Registry, a large observational cohort published by Frogel et al. (2008), included ≈19,548 infants who received at least one dose of palivizumab, showing an RSV hospitalization rate of 1.6% among treated infants, indicating the effectiveness in clinical practice [29]. In a retrospective cohort of 415 preterm infants (<32 weeks’ gestation), palivizumab prophylaxis was associated with a 90% reduction in RSV-associated hospitalizations during the first 6 months after birth discharge (adjusted odds ratio 0.1, 95% CI 0.01–0.9; *p* = 0.01). This protective effect was no longer statistically significant from 7 to 12 months after discharge (50% reduction; *p* = 0.51) [30]. Palivizumab prophylaxis was associated with a significant decrease in RSV hospitalizations in moderate-to-late preterm infants (29–35 weeks’ gestation) according to a systematic review of six real-world cohort studies. The weighted mean indicated a roughly 4-fold decrease compared with untreated infants, which closely matched reductions seen in RCTs [31]. The summary of the studies is found in Table 1.

#### 3.1.2. Nirsevimab Efficacy

The clinical efficacy of nirsevimab has been demonstrated across multiple randomized controlled trials. In the HARMONIE trial published by Drysdale et al. (2019; NCT05437510) that enrolled 8058 infants entering their first RSV season, a single dose of nirsevimab reduced hospitalizations for RSV-associated lower respiratory tract infection (LRTI) by 83.2% compared with standard care (0.3% vs. 1.5%; 95% CI, 67.8–92.0; *p* < 0.001). The incidence of very severe RSV-associated LRTI was also reduced by 75.7% (0.1% vs. 0.5%; 95% CI, 32.8–92.9). Efficacy was consistent across participating countries, including France (89.6%), Germany (74.2%), and the United Kingdom (83.4%) [27]. A trial published by Griffin et al. in 2020 (NCT02878330) involving 1453 infants (969 receiving nirsevimab vs. 484 placebo group) demonstrated a 70.1% reduction in medically attended RSV-associated LRTI compared to placebo (2.6% vs. 9.5%; 95% CI: 52.3–81.2%; *p* < 0.001). RSV-related hospitalizations were similarly reduced by 78.4% (0.8% vs. 4.1%; 95% CI: 51.9–90.3%) [21]. In 2022, Hemmit et al. published the MELODY phase 2b/3 trial (NCT03959488) that enrolled 1490 late-preterm and term newborns in their first year of life. In this study, nirsevimab reduced medically attended RSV-associated LRTI by 74.5% compared with placebo (1.2% vs. 5.0%; 95% CI, 49.6–87.1; *p* < 0.001), while RSV-related hospitalizations were reduced by 77.3% (0.6% vs. 2.6%; 95% CI, 32.3–93.0%) [32]. Following the introduction of nirsevimab into global immunization programs, actual data have revealed significant decreases in RSV hospitalizations [33,34,35,36]. These data confirm the results of the clinical trial and reveal efficacy in more general settings. The largest retrospective cohort we found was published by Pelletier et al. (2025), and it consisted of 409,723 infants born during the 2024–2025 RSV season. Infants who received nirsevimab had significantly lower rates of RSV-associated hospitalization compared with untreated infants (0.4% vs. 1.2%; *p* < 0.001) and a shorter median length of hospital stay. Nirsevimab was associated with a significantly lower risk of RSV hospitalization (adjusted hazard ratio 0.23, 95% CI 0.21–0.26) after adjustment for demographic and clinical covariates. RSV-associated intensive care unit (ICU) admissions were also significantly lower among infants who received nirsevimab compared with untreated infants (0.2% vs. 0.4%, *p* < 0.001), corresponding to an approximate 50% relative risk reduction [33]. In a retrospective real-world evaluation of 1156 newborns in Tyrol, Austria, implementation of a regional nirsevimab immunization program was associated with a significant decrease in RSV-related hospitalizations (151 vs. 47; *p* = 0.018) and shorter lengths of hospital stay compared with pre-implementation seasons. None of the infants hospitalized during the post-implementation period had received nirsevimab [37]. In a large multicenter case–control study conducted across 27 pediatric hospitals in the United States, administration of nirsevimab at least 7 days prior to respiratory symptom onset was associated with an estimated 80% effectiveness (95% CI: 70–86%) against RSV-associated ICU (intensive care unit) admission and *83% effectiveness (95% CI: 74–90%) against acute respiratory failure among infants admitted to ICU during their first RSV season. The efficacy was highest (86%, 95% CI: 77–92%) at 7–59 days after dose and lower (66%, 95% CI: 47–79%) beyond 60 days, supporting the sustained protective effect of nirsevimab against severe RSV outcomes in real-world settings through time [38]. Consistent findings were observed in a large test-negative case–control study of 3090 infants within the Yale New Haven Health System in the United States. After adjustment for potential confounders, nirsevimab effectiveness was estimated at 68.4% (95% CI, 50.3–80.8%) against medically attended RSV infection, 80.5% (95% CI, 52.0–93.5%) against RSV-associated hospitalization, and 84.6% (95% CI, 58.7–95.6%) against severe RSV disease. Effectiveness was highest shortly after immunization (79.3% at two weeks) and gradually declined over time, reaching 54.8% by 14 weeks post-administration [39]. The most recent study worth mentioning was a region-wide implementation study from Lombardy, Italy, which reported that following a universal immunization campaign with nirsevimab in infants <12 months during the 2024–2025 RSV season, emergency department visits for acute lower respiratory infections decreased by approximately 43% and hospitalizations by approximately 47%. Following the implementation, emergency department visits for acute lower respiratory infections declined by approximately 43%, and hospitalizations decreased by approximately 47%. RSV-specific emergency department visits and hospitalizations were reduced by approximately 49% and 55%, respectively, compared with historical trends. Importantly, no comparable reductions were observed among older children (>12 months) who were not eligible for immunization, supporting a population-level impact of nirsevimab prophylaxis [40]. The summary of the studies is found in Table 2.

#### 3.1.3. Head-to-Head Comparison

No direct head-to-head in vivo comparisons between nirsevimab and palivizumab have been reported in the literature. The analysis of the MEDLEY randomized trial demonstrated that a single dose of nirsevimab in infants at risk of severe RSV infection induced RSV-neutralizing antibody (nAb) levels approximately 10-fold higher than those achieved with monthly palivizumab, with levels remaining elevated for up to one-year post-administration. Peak nAb levels were observed shortly after dosing and remained well above baseline at 360 days. Furthermore, serum concentrations of nirsevimab were strongly correlated with RSV nAb levels, suggesting the possibility for longer protection against RSV in the general population rather than only in high-risk populations [43].

### 3.2. Safety Profile Studies

#### 3.2.1. Palivizumab Safety

Palivizumab has an established safety profile with over 25 years of clinical use [44]. The most common adverse events are mild and include injection site reactions, fever, and rash (occurring in 2–3% of patients) [45,46]. Serious adverse events are rare, with incidence rates comparable to placebo in controlled trials [15,28,45,47,48], while anaphylactic reactions are uncommon (fewer than 0.1% of cases) [49]. Data from a large expanded-access safety study in the Northern Hemisphere confirmed that palivizumab is generally well tolerated, with no unexpected safety concerns. The adverse events observed were consistent with those typically associated with injections and common pediatric symptoms [50]. An open-label safety trial evaluating repeated seasonal prophylaxis found no increase in adverse reactions with successive doses, reinforcing its safety for extended use [51]. Post-marketing surveillance data covering millions of doses administered worldwide confirm palivizumab’s safety profile, with no new safety signals identified [52,53]. The summary of the studies is found in Table 3.

#### 3.2.2. Nirsevimab Safety

Nirsevimab’s safety profile has been evaluated in over 4000 infants across clinical trials [21,32]. In the clinical trial published by Griffin et al., nirsevimab demonstrated a safety profile comparable to placebo, with serious adverse events reported in 11.2% of nirsevimab recipients versus 16.9% of placebo recipients, and grade ≥3 events occurring in 8.0% versus 12.5%, respectively. Most adverse reactions were mild to moderate, with rash and petechiae reported in 0.5% of nirsevimab recipients versus 0.6% of placebo recipients [21]. In the MELODY study by Hammit et al., the safety profile of nirsevimab was comparable to that of placebo, with mostly mild to moderate adverse events; grade ≥3 events occurred in 3.6% of nirsevimab recipients versus 4.3% of placebo recipients, and serious adverse events in 6.8% versus 7.3%, respectively. Reported reactions included pyrexia, discomfort, injection-site pain or swelling, vomiting, hypoglycemia, anemia, and a single case of grade 3 generalized macular rash considered related to nirsevimab [32]. Neither trial reported anaphylactic reactions or deaths attributable to nirsevimab [21,32]. Based on safety and tolerability data from Domachowske et al. (2022), nirsevimab had a safety profile comparable to palivizumab in high-risk infants, including those with congenital heart disease, chronic lung disease, or prematurity. There were low rates of medically attended RSV LRTI, no new safety signals, and no deaths related to the antibody [54]. Real-world safety data from national immunization programs across Europe have confirmed these results, showing adverse event rates consistent with those observed in clinical trial data [55,56,57,58]. The summary of the studies is found in Table 4.

### 3.3. Cost-Effectiveness Analyses

#### 3.3.1. Palivizumab Cost-Effectiveness

A U.S. decision-analytic cost-effectiveness analysis evaluating palivizumab prophylaxis in premature infants without chronic lung disease concluded that prophylaxis was not cost-effective compared with no prophylaxis. Incremental cost-effectiveness ratios (ICERs) varied widely by gestational age, ranging from approximately USD 675,780 to more than USD 1,850,000 per QALY gained. These findings suggest that the additional costs associated with palivizumab substantially exceeded the potential health benefits and cost savings related to reduced RSV hospitalizations or long-term respiratory sequelae such as asthma [59]. In contrast, a Canadian decision-analytic model focusing on infants born at 32–35 weeks’ gestation without chronic lung disease reported higher overall costs but modest gains in quality-adjusted life years with palivizumab prophylaxis, resulting in an ICER of CAD $20,924 per QALY gained. This value was considered cost-effective within the Canadian healthcare context. The results were robust in sensitivity analyses, with a 99% probability that the ICER remained below $50,000 per QALY. The cost-effectiveness analyses were the most favorable among infants with two or more risk factors, with ICERs ranging from CAD 808 to CAD 81,331 per QALY, supporting targeted prophylaxis in moderate to high-risk populations [60]. Also, a study that analyzes the cost-effectiveness in the Eastern Canadian Arctic found that using palivizumab to prevent RSV was worth the cost for Inuit infants at high risk of getting the infection. When region-specific hospitalization costs and local epidemiologic data were incorporated, ICERs fell below commonly accepted willingness-to-pay thresholds, underscoring the importance of contextual factors in economic evaluations of RSV prophylaxis [61]. A Spanish cost-effectiveness analysis evaluating palivizumab prophylaxis in premature infants (32–35 wGA) with two or more risk factors reported incremental cost-effectiveness ratios (ICERs) ranging from approximately €6142 to €12,814 per quality-adjusted life year (QALY) gained. These values were well below the commonly cited Spanish willingness-to-pay threshold of €30,000 per QALY. Inclusion of indirect costs. When indirect costs were incorporated, palivizumab prophylaxis became cost-saving from a societal perspective, highlighting its economic efficiency in this high-risk European setting [62]. Similarly, an Austrian cost-effectiveness analysis based on nationwide epidemiologic data found palivizumab prophylaxis to be cost-effective in preventing RSV disease among high-risk infants. ICERs per QALY gained varied across subgroups, ranging from €8484 to €26,292, but generally remained within accepted cost-effectiveness thresholds in Austria [63]. In the UK, a cost-effectiveness model by Narayan et al. (2020) demonstrated that palivizumab prophylaxis was dominant (i.e., more effective and cost-saving) compared with no prophylaxis across multiple high-risk infant subgroups. Among infants with congenital heart disease (CHD), prophylaxis was associated with cost reductions of £13,689 and gains of 4 QALYs, with an incremental net monetary benefit (INMB) of £112,989. In infants with bronchopulmonary dysplasia (BPD), costs decreased by £10,812 with 4 additional QALYs (INMB £122,797). Palivizumab was also dominant in premature infants younger than six months without congenital heart disease or bronchopulmonary dysplasia, including those born at <29 weeks’ gestation (£133,889 saved; 3 QALYs gained; INMB £221,881), 29–32 weeks’ gestation (£59,834 saved; 2 QALYs gained; INMB £126,919), and 33–35 weeks’ gestation (£55,314 saved; 2 QALYs gained; INMB £121,124) [64]. Finally, a Canadian cost-utility analysis employing the International Risk Scoring Tool (IRST) and the Canadian Risk Scoring Tool (CRST) to guide palivizumab prophylaxis in moderate-to-late preterm infants (32–35 wGA) reported ICERs of CAD 29,789 per QALY gained using IRST and CAD 15,833 per QALY gained using CRST. Both approaches demonstrated a high probability of cost-effectiveness at a willingness-to-pay threshold of CAD 50,000 per QALY, with vial sharing further enhancing cost-utility in sensitivity analyses [65]. The summary of the studies is found in Table 5.

#### 3.3.2. Nirsevimab Cost-Effectiveness

Economic evaluations of nirsevimab for the prevention of RSV in infants reveal heterogeneity in cost-effectiveness profiles. It really depends on the dose price, target population, and modeling assumptions. In a U.S. birth cohort model, nirsevimab was projected to prevent a substantial number of RSV-related outcomes, including hospitalizations, with an estimated incremental cost-effectiveness ratio (ICER) of approximately USD 153,517 per QALY gained. The results demonstrated considerable variability, ranging from cost-saving scenarios to ICERs exceeding USD 323,000 per QALY, depending on assumptions related to hospitalization costs, clinical effectiveness, and the duration of antibody-mediated protection. Cost-effectiveness was consistently more favorable when analyses focused on higher-risk infant populations [66]. In England and Wales, analysis suggested that nirsevimab could represent a cost-effective alternative to the palivizumab program, due to the low cost per dose (≤£63), so there were adopted seasonal administration strategies were adopted. Lower price thresholds were required to achieve cost-effectiveness when extending coverage to broader infant populations [67]. A Canadian cost-effectiveness analysis reported that, at current baseline prices, nirsevimab strategies (especially when targeted to high-risk or preterm infants) were both more effective and cost-saving compared with no intervention in several regions. The results showed that universal infant immunization would require a per-dose price below approximately CAD 112 to be considered the most cost-effective strategy at the national level [68]. In Spain, modeling studies comparing nirsevimab versus standard practice incorporated acquisition costs, healthcare resource utilization, and indirect costs to estimate economically justifiable pricing thresholds for RSV prevention. These analyses underlined that cost-effectiveness is very sensitive to local infection rates, healthcare costs, and assumptions about the duration of antibody protection.

Several economic evaluations have suggested that nirsevimab could be cost-effective for infant RSV prevention in certain price ranges. A single study found nirsevimab is likely worth the investment if it is priced close to €220 per dose [69]. An Italian cost-utility model found that prophylaxis with nirsevimab was cost-effective across a range of willingness to pay thresholds (€22,000–€30,000 per QALY) for an all-infants strategy. The model also found price ranges considered economically justifiable were approximately €267–€400 per QALY gained, while higher price thresholds were found when indirect costs were considered, reflecting broader societal benefits [70]. A Dutch economic evaluation using a static cost-effectiveness model found that universal infant immunization with nirsevimab would be considered cost-effective at a willingness to pay threshold of €50,000 per QALY, based on an acquisition price of approximately €220 per dose. Compared to standard care, the all-infants strategy resulted in substantial reductions in RSV-related cases, hospitalizations, and intensive care unit admissions [71]. In Japan, an economic model demonstrated that universal nirsevimab immunization was cost-effective at a willingness-to-pay threshold of ¥5,000,000 per QALY. The base-case analysis resulted in an ICER of ¥4,537,256 per QALY gained, which was improved to ¥1,695,635 per QALY when analyzed from a societal perspective. The model estimated a 50% reduction in RSV-associated health events in infants compared with standard care [72]. Finally, in a Chinese economic model study using a Markov decision-tree approach, both seasonal and year-round nirsevimab immunization strategies were found to be cost-effective compared with no prophylaxis in infants in Shanghai when analyzed using a willingness-to-pay threshold equivalent to GDP per capita. Seasonal administration was associated with lower ICERs than continuous year-round immunization, indicating the impact of program design on economic evaluations [73]. The summary of the studies is found in Table 6.

Table 7 summarizes country-specific willingness-to-pay thresholds and acquisition price ranges at which nirsevimab was considered cost-effective.

## 4. Discussion

This systematic review tried to synthesize the available evidence on the efficacy, safety profile, and cost-effectiveness of palivizumab and nirsevimab for the prevention of RSV in the pediatric population. Our findings indicate that while both monoclonal antibodies offer meaningful protection against RSV-associated hospitalizations, they differ substantially in terms of clinical use, implementation, and economic impact.

### 4.1. Efficacy and Clinical Impact

Palivizumab has shown consistent efficacy in reducing RSV-related hospitalizations, with relative risk reductions of approximately 45–55% in high-risk pediatric populations across pivotal clinical trials and real-world studies. These effects have been consistently supported by multiple systematic reviews and meta-analyses showing palivizumab’s established role in targeted RSV prophylaxis. However, current evidence indicates that palivizumab does not significantly reduce mortality, length of hospital stays, or other adverse clinical outcomes beyond the prevention of hospitalization. There is a general limitation in the scope of this preventive effect, such as insufficient evidence to support the routine use of palivizumab in high-risk conditions, such as cystic fibrosis, thus necessitating further research in populations with comorbidities beyond prematurity, congenital heart disease, or chronic lung disease. There are logistical challenges associated with the requirement for monthly injections throughout the RSV season. There is a general limitation in the scope of palivizumab’s clinical effectiveness, such as a lack of effect on a vast majority of RSV disease burden, especially in healthy term infants [11,74,75,76]. Nirsevimab has demonstrated a high level of relative efficacy in preventing RSV-associated lower respiratory tract infections and hospitalizations, with risk reductions ranging from 70 to 80% across randomized clinical trials and real-world studies. Importantly, there is a high level of protection shown by nirsevimab among a wide range of infants, such as healthy term and late-preterm infants, during their first RSV season. Evidence from large pragmatic trials and population-based studies from Europe and North America indicates that these clinical benefits translate into real-world effectiveness at scale, with substantial reductions in hospital admissions, ICU utilization, and emergency department visits [25,43,77,78,79].

### 4.2. Safety Profile

Both palivizumab and nirsevimab demonstrate favorable safety profiles consistent with their mechanisms as passive immunoprophylactic agents. More than 25 years of clinical experience and extensive post-marketing surveillance covering millions of administered doses worldwide are particularly advantageous for palivizumab. About 2–3% of recipients experience reported adverse events, which are predominantly mild and include injection-site reactions, fever, and rash. While anaphylactic reactions are extremely rare (less than 0.1%), serious adverse events have been reported at rates similar to placebo in controlled clinical trials [15,47,48,49,50,51]. Long-term safety studies assessing repeat-season administration have demonstrated no increase in adverse reactions with repeated exposure, supporting the tolerability of palivizumab across multiple RSV seasons [52,53].

Nirsevimab’s safety profile has been established through clinical trials involving over 4000 infants and real-world surveillance programs across multiple countries. The MELODY and HARMONIE trials reported adverse event rates comparable to or lower than placebo, with serious adverse events occurring in 6.8% of nirsevimab recipients versus 7.3% of placebo recipients [32,41]. In post-marketing surveillance carried out in various countries such as Spain, Luxembourg, Western Australia, and Italy, no new safety concerns have been established for nirsevimab. In addition, most adverse events have been mild and transient [55,56,57,58]. Comparative data from the MEDLEY trial further indicate that nirsevimab exhibits a safety and tolerability profile similar to that of palivizumab in high-risk infants, including those with congenital heart disease or chronic lung disease. These results support the use of nirsevimab not only in healthy term and preterm infants but also in vulnerable populations traditionally targeted for palivizumab prophylaxis [54].

### 4.3. Cost-Effectiveness

The economic evaluation of RSV immunoprophylaxis reveals substantial heterogeneity across healthcare systems, target populations, and methodological approaches. Palivizumab is generally found to be cost-effective in narrowly defined high-risk groups, particularly in settings with elevated RSV hospitalization rates or high inpatient costs. However, many analyses report very high incremental cost-effectiveness ratios (ICERs) that exceed commonly accepted willingness-to-pay thresholds when prophylaxis is extended to broader infant populations. This heterogeneity largely reflects the high acquisition cost of palivizumab, the requirement for multiple monthly doses, and the relatively modest absolute reductions in hospitalizations among lower-risk infants [80,81,82,83,84]. In contrast, cost-effectiveness assessments of nirsevimab suggest a more favorable economic profile, although outcomes remain highly sensitive to price, implementation strategies, and local epidemiologic conditions.

Studies from Europe, Canada, and Asia indicate that nirsevimab can be cost-effective when it targets high-risk or preterm infants, or when administered seasonally in alignment with peak RSV circulation. Universal infant immunization programs can provide a significant reduction in RSV-related healthcare utilization. However, their cost-effectiveness depends on maintaining acquisition prices below certain levels to stay within the willingness-to-pay limits. Overall, the evidence suggests that nirsevimab has the potential to improve the economic efficiency of RSV prevention compared to palivizumab by reducing the need for repeated dosing, protecting the infant populations that are responsible for the most RSV hospitalizations, and facilitating scalable public health programs. Nonetheless, there are still uncertainties related to long-term pricing, duration of antibody-mediated protection, and indirect effects on RSV transmission. It is suggested that economic evaluation should be continued with the availability of real-world data [85,86,87]. Palivizumab has been used for RSV prophylaxis of infants with narrowly defined conditions of high-risk (e.g., extreme prematurity, chronic lung disease, or significant congenital heart disease) requiring multiple doses throughout the RSV season. In contrast, nirsevimab offers broader indications and is recommended for nearly all infants about to enter their first RSV season due to its long half-life and the convenience of a single-dose protection.

### 4.4. Maternal RSV Vaccination as an Alternative Preventive Strategy

Maternal RSV vaccination has emerged as an additional preventive strategy aimed to protect infants during the early postnatal period through the transplacental transfer of antibodies [84]. In the phase 3 trial evaluating the Respiratory Syncytial Virus Prefusion F (RSVpreF) vaccine, immunization during late pregnancy was associated with an approximate 81.8% reduction in severe RSV-associated lower respiratory tract illness among infants within the first 90 days of life, with evidence of waning protection thereafter [88]. This early window of protection represents a meaningful advantage, particularly for infants born before postnatal prophylaxis programs can be initiated.

However, several important limitations affect the real-world applicability of maternal vaccination. The durability of protection is limited: antibody levels transferred transplacental decline over the first months of life, and protection beyond 90–180 days decreases substantially, leaving infants unprotected during the latter part of the RSV season [88,89]. The RSV vaccination must occur within a specific gestational window (32–36 weeks) to allow adequate antibody transfer before delivery, which creates logistical challenges in routine antenatal care. Maternal vaccine uptake is subject to the same hesitancy and access barriers that affect other antenatal interventions, and coverage in real-world programs may fall short of trial conditions [90].

Available data suggest that nirsevimab offers a longer duration of protection within a single RSV season and can be administered regardless of maternal vaccination status, making the two strategies potentially complementary rather than mutually exclusive [91]. A large population-based cohort study conducted in France (Jabagi et al., 2025), enrolling over 42,000 infants, found that nirsevimab was associated with a 26% lower risk of RSV-related hospitalization compared with maternal RSVpreF vaccination (aHR 0.74; 95% CI 0.61–0.88), with additional reductions in ICU admission rates and need for ventilatory support [92]. A multicenter surveillance study in the United States (Moline et al., 2025) reported effectiveness against RSV-associated hospitalization of 81% for nirsevimab and 70% for maternal vaccination across seven pediatric centers, with overall hospitalization rates declining by up to 51% compared with pre-intervention seasons. These findings suggest that, while both strategies confer meaningful protection, nirsevimab may offer broader and more consistent coverage across the full RSV season [93].

The two strategies are not necessarily mutually exclusive: given that maternal vaccination offers protection from birth while nirsevimab is administered postnatally, a combined approach may provide the most comprehensive coverage in settings where both are available and affordable. The cost-effectiveness and feasibility of such combined strategies, however, require further evaluation before broad recommendations can be made.

### 4.5. Parent Compliance and Acceptance of Vaccine

The effectiveness of RSV immunization programs is influenced not only by clinical efficacy and cost-effectiveness but also by parental acceptance and real-world uptake. For example, the requirement for monthly administration of palivizumab throughout the RSV season (typically involving five doses) is a substantial challenge and has been associated with suboptimal adherence. Indeed, several studies report that only 22–44.5% of eligible infants receive the full recommended course of prophylaxis [94,95]. In contrast, reported acceptance of nirsevimab has been higher, although variable across settings, ranging from 91.6% in France to 60% in Canada and 57% in Austria [37,96,97].

Vaccine hesitancy remains a potential barrier to the successful implementation of RSV prevention strategies, especially in areas where there is a lack of information on monoclonal antibodies or other recently introduced measures. Evidence consistently indicates that recommendations from healthcare providers are the strongest predictors of parental acceptance. All of this highlights the need for education and effective communication. In recent years, and especially after the COVID-19 pandemics vaccine misinformation and hesitancy have increased, leading to outbreaks of vaccine-preventable disease, including measles and pertussis, in different areas of the world [98,99,100,101,102,103,104,105]. Another good example is the suboptimal seasonal vaccination rate for influenza reported in children [106,107,108]. A real-life program should include monitoring of vaccine uptake, reasons for refusal, and addressing parental concerns.

### 4.6. Limitations and Future Research Directions

Our systematic review has several limitations. The absence of head-to-head clinical trials comparing nirsevimab and palivizumab necessitated reliance on indirect comparisons from studies conducted in different populations and across distinct time periods. Heterogeneity in study design, definitions, and populations limits the ability to make definitive conclusions on their relative efficacy. Cost-effectiveness analyses are highly context-dependent and remain sensitive to assumptions related to long-term outcomes, duration of protection, and potential indirect benefits. In addition, the majority of available evidence on nirsevimab originates from high-income settings, while evidence from low- and middle-income countries remains limited. The economic evaluations of palivizumab and nirsevimab were conducted in different countries, across different time periods (2006–2025), using different currencies and different target populations. No currency conversion or inflation adjustment was applied, as doing so would require specifying a reference year and exchange rate assumptions not present in the source publications, introducing additional uncertainty. Accordingly, cost-effectiveness data for nirsevimab and palivizumab are presented separately and descriptively, and cross-study comparisons should be interpreted with caution.

The following areas should be addressed in future research. Long-term follow-up studies are needed to determine whether RSV prevention during infancy influences subsequent respiratory outcomes, including the development of asthma and recurrent wheezing. The optimal integration of maternal vaccination strategies with infant immunoprophylaxis requires further investigation through comparative effectiveness studies. The evaluation of the economic impact of RSV infection should be extended to include societal considerations, including the impact on parental productivity. Real-world effectiveness studies conducted across diverse geographic regions and healthcare systems will be essential to better understand the impact of nirsevimab in different epidemiologic conditions of RSV infection. The importance of surveillance after marketing cannot be overstated, particularly regarding rare side effects that may not be evident in clinical trials.

Recently, on 9 June 2025 FDA approved clesrovimab, another long-acting monoclonal antibody for RSV prophylaxis, representing an additional promising option for RSV prevention strategies [109]. Recent clinical trial data indicate that clesrovimab provides substantial protection against RSV-associated lower respiratory tract infection and hospitalization in infants, with a favorable safety profile and no major safety signals reported compared with placebo [110,111].

## 5. Conclusions

Nirsevimab represents a promising advance in RSV prevention in infants and young children, with a favorable efficacy and safety profile and a single-dose administration schedule that offers practical advantages over palivizumab in terms of programmatic feasibility and population coverage. Across the studies included in this review, nirsevimab demonstrated higher efficacy estimates in pivotal trials enrolling healthy term and late-preterm infants, a population for which palivizumab is not routinely indicated, and real-world data from multiple countries support these findings in clinical practice.

However, there are important cautions that should be acknowledged. No direct head-to-head trial comparison between nirsevimab and palivizumab currently exists, and the indirect nature of available comparisons limits the strength of conclusions that can be drawn. The cost-effectiveness of nirsevimab is highly sensitive to acquisition price, target population, healthcare system context, and willingness-to-pay thresholds, and is not uniformly established across all settings. Palivizumab retains a well-established role in high-risk populations, particularly preterm infants, and those with congenital heart disease or chronic lung disease, where decades of clinical experience and a robust safety record support its continued use, especially in settings where nirsevimab availability or affordability remains limited.

The accumulated evidence supports further evaluation of nirsevimab-based strategies for broader RSV prevention, including universal infant immunization programs, where pricing and health system conditions allow. The development of context-specific, price-sensitive recommendations will be essential for converting the potential of nirsevimab into an equitable public health impact. Ultimately, the choice between available RSV prophylaxis strategies should be guided by local epidemiology, healthcare resources, and the best available evidence at the time of decision-making.

## Figures and Tables

**Table 1:** Summary of palivizumab efficacy studies.

Study (Author, Year)	Design/Sample Size	Population	Intervention	Key Findings	Newcastle-Ottawa Scale/Cochrane RoB
The IMpact-RSV Study Group, 1998 [15]	RCT/N = 1502 (1002 palivizumab; 500 placebo)	Premature infants ≤35 weeks GA or BPD	Palivizumab 15 mg/kg monthly × 5 doses vs. placebo	55% reduction in RSV hospitalizations (4.8% vs. 10.6%, *p* < 0.001). NNT = 17. No mortality difference.	Low risk of bias
Feltes et al., 2003 [28]	RCT/N = 1287 (639 palivizumab; 648 placebo)	Children with hemodynamically significant CHD	Palivizumab 15 mg/kg monthly × 5 doses vs. placebo	45% reduction in RSV hospitalizations (5.3% vs. 9.7%, *p* < 0.003). Reduced total hospital days by 73% (*p* = 0.14).	Low risk of bias
Frogel et al., 2008 [29]	Cohort/N = 19,548 (palivizumab recipients)	High-risk infants receiving palivizumab	Palivizumab monthly during RSV season	Palivizumab prophylaxis was associated with a low RSV hospitalization rate of 1.6% among high-risk infants.	Moderate risk of bias
Yeo et al., 2021 [30]	Retrospective cohort/N = 415 (109 palivizumab; 306 no prophylaxis)	Preterm infants <32 wks GA	Palivizumab vs. no prophylaxis	RSV hospitalization 2.8% vs. 10.5% (*p* = 0.02). Adjusted 90% reduction in RSV hospitalizations at 0–6 months (aOR 0.1, 95% CI 0.01–0.9, *p* = 0.01); not significant 7–12 mo (50%, *p* = 0.51).	Moderate risk of bias
Manzoni et al., 2022 [31]	Systematic review/6 cohort studies	Preterm infants 29–35 wks GA	Palivizumab vs. no prophylaxis	Weighted mean ~4-fold reduction in RSV hospitalization; findings consistent with RCT efficacy.	Moderate risk of bias

Abbreviations: RSV—respiratory syncytial virus; RCT—randomized controlled trial; GA—gestational age; BPD—bronchopulmonary dysplasia; CHD—congenital heart defects.

**Table 2:** Summary of nirsevimab efficacy studies.

Study (Author, Year)	Design/Sample Size	Population	Intervention	Key Findings
Griffin et al., 2020 [21]	RCT/N = 1453 (969 nirsevimab; 484 placebo)	Healthy preterm infants (29–35 weeks GA) entering the first RSV season	Nirsevimab single dose vs. placebo	Nirsevimab reduced medically attended RSV-associated LRTI by 70.1% (2.6% vs. 9.5%; 95% CI 52.3–81.2; *p* < 0.001) and RSV-related hospitalizations by 78.4% (0.8% vs. 4.1%; 95% CI 51.9–90.3)
Hammitt et al., 2022 (MELODY) [32]	RCT/N = 1490 (994 nirsevimab; 496 placebo)	Healthy late-preterm and term infants entering the first RSV season	Nirsevimab single dose (50 mg <5 kg; 100 mg ≥5 kg) vs. placebo	74.5% reduction in medically attended RSV-LRTI (1.2% vs. 5.0%, *p* < 0.001). 62.1% reduction in RSV hospitalizations (0.6% vs. 1.6%, *p* = 0.07)
Drysdale et al., 2023 (HARMONIE) [41]	Pragmatic RCT/N = 8058 (4037 nirsevimab; 4021 no RSV prophylaxis)	Healthy infants ≥ 29 weeks’ gestation entering their first RSV season	Nirsevimab single dose vs. standard care (no RSV prophylaxis)	Hospitalization for RSV-associated LRTI was significantly reduced (0.3% vs. 1.5%; efficacy 83.2%; *p* < 0.001); very severe RSV-LRTI was reduced (0.1% vs. 0.5%; efficacy 75.7%, *p* = 0.004)
Ezpeleta et al., 2024 [35]	Observational population cohort/N = 1177 infants (1083 nirsevimab; 94 non-immunized)	Healthy infants (they did not stratify by gestational age) (Spain)	Nirsevimab immunoprophylaxis at birth vs. no immunization	RSV-related hospitalization was 0.7% in nirsevimab recipients vs. 8.5% in non-immunized infants, with estimated effectiveness 88.7% (95% CI, 69.6–95.8) in preventing RSV-associated hospitalization; ICU admission also reduced (0.3% vs. 2.1%)
Rodríguez-Fernández et al., 2024 [34]	Observational population cohort	Infants <6 months (pre-nirsevimab period vs. nirsevimab period) (Spain)	Nirsevimab implementation (universal prophylaxis) vs. historical control (no nirsevimab)	Hospital admissions for RSV bronchiolitis in infants <6 months decreased from 574 of 1195 (48%) in pre-nirsevimab seasons to 6 of 138 (4.3%) after nirsevimab implementation (*p* < 0.01), corresponding to an estimated effectiveness of 85% (95% CI 32–97%)
Moline et al., 2024 [42]	Cohort/N = 699 (407 nirsevimab; 292 no prophylaxis)	Infants <8 months in the US	Nirsevimab single dose vs. no immunization	Effectiveness against RSV-associated hospitalization: 90% (95% CI 75–96%) among infants receiving nirsevimab ≥7 days before symptom onset; median time from dose to symptom onset 45 days (IQR 19–76)
Pelletier et al., 2025 [33]	Retrospective cohort/N = 409,723 (194,422 nirsevimab; 215,301 no prophylaxis)	Infants born during the 2024–2025 RSV season (multicountry study)	Nirsevimab administration vs. no nirsevimab	RSV hospitalization 0.4% in the nirsevimab group vs. 1.2% no prophylaxis group (*p* < 0.001); adjusted HR for RSV hospitalization 0.23 (95% CI 0.21–0.26). The rate of ICU admissions was also significantly lower among infants who received nirsevimab compared with untreated infants (0.2% vs. 0.4%, *p* < 0.001)
Höck et al., 2025 [37]	Retrospective observational cohort/N = 1156 newborns	Infants born in 3 maternity wards in Tyrol, Austria	Nirsevimab immunization program (57% coverage)	Post-implementation RSV hospitalizations decreased from 151 to 47 (*p* = 0.018); median age at admission was higher, and length of stay was shorter after implementation; no hospitalized infants had received nirsevimab
Zambrano et al., 2025 [38]	Multicenter case–control/N = 759 infants (457 case-patients; 302 controls)	Infants <1 year admitted to ICU with respiratory symptoms (USA)	Nirsevimab ≥7 days before symptom onset vs. no nirsevimab	Nirsevimab was 80% effective (95% CI: 70–86%) against RSV-associated ICU admission and 83% effective (95% CI: 74–90%) against acute respiratory failure; effectiveness was highest 7–59 days after dose (86%) vs. 60–183 days (66%)
Xu et al., 2025 [39]	Test-negative case–control/N = 3090 infants	Infants tested for RSV (USA)	Nirsevimab immunization vs. no immunization	Adjusted effectiveness 68.4% against medically attended RSV infection, 80.5% against RSV hospitalization, and 84.6% against severe RSV disease; effectiveness waned over time (79.3% at 2 weeks to 54.8% at 14 weeks)
Villa et al., 2026 [40]	Region-level interrupted time-series, infants <12 months, Lombardy Region, Italy	Infants born during the 2024–2025 RSV season (Italy)	Universal nirsevimab immunization campaign	Emergency visits for LRTI decreased by 42.7%; hospitalizations decreased by 46.5%; RSV-associated EDVs decreased by 49.3%; RSV hospitalizations decreased by 55.0% post-campaign vs. historical trends

Abbreviations: RSV—respiratory syncytial virus; RCT—randomized controlled trial; GA—gestational age; LRTI—lower respiratory tract infections; ICU—intensive care unit.

**Table 3:** Summary of Palivizumab safety.

Study (Author, Year)	Design/Sample Size	Population	Intervention	Key Findings	Newcastle-Ottawa Scale/Cochrane RoB
The IMpact-RSV Study Group, 1998 [15]	RCT/N = 1502 (1002 palivizumab; 500 placebo)	Premature infants ≤35 weeks GA or BPD	Palivizumab 15 mg/kg monthly × 5 doses vs. placebo	No significant differences in adverse events between palivizumab and placebo, low rates of injection-site reactions (2.7% vs. 1.8%), few discontinuations for related adverse events (0.3%), and similar hepatic and renal adverse event rates across groups.	Low risk of bias
Meissner et al., 1999 [47]	Randomized, double-blind, placebo-controlled trial/N = 43 (33 palivizumab; 10 placebo)	Infants and young children at risk for severe RSV disease	palivizumab 2 doses vs. placebo	Palivizumab was safe and well-tolerated with expected serum levels	Low risk of bias
Groothuis JR, 2001 [50]	Expanded access safety trial/N = 565	High-risk infants receiving palivizumab	Palivizumab IM monthly	Palivizumab administration was well tolerated with no unexpected safety signals; minor injection site reactions and common infant AEs were reported, consistent with the known safety profile	Moderate risk of bias
Groothuis JR, 2003 [48]	Multicenter safety cohort/N = 285	Preterm infants 29–32 weeks’ gestation without CLD	Palivizumab 15 mg/kg IM monthly	Palivizumab was safe and well-tolerated; common AEs included rhinitis, cough, fever, pharyngitis, bronchiolitis, diarrhea; no deaths reported	Moderate risk of bias
Lacaze-Masmonteil et al., 2003 [51]	Open-label safety trial/N = 134 (71 first season, 63 s season)	Infants receiving palivizumab for >1 season	Palivizumab 15 mg/kg IM monthly	No increase in adverse reactions with repeat exposure	Moderate risk of bias
Feltes et al., 2003 [28]	RCT/N = 1287 (639 palivizumab; 648 placebo)	Children with hemodynamically significant CHD	Palivizumab 15 mg/kg monthly × 5 doses vs. placebo	Serious adverse events occurred in 55.4% of the palivizumab group versus 63.1% of the placebo group; 3.3% versus 4.2% deaths, respectively. No event or death was attributed to palivizumab	Low risk of bias
Kashiwagi et al., 2017 [52]	Multicenter post-marketing surveillance/N = 304	Children ≤24 mo with immunocompromised conditions or Down syndrome	Palivizumab prophylaxis	Palivizumab is generally safe with modest adverse events	Moderate risk of bias
Castillo et al., 2017 [53]	Prospective observational cohort/N = 458	Infants at risk for severe RSV infection	Palivizumab prophylaxis	1165 adverse events were recorded during one year of follow-up, with 135 serious adverse events, but no events were considered to be related to palivizumab.	Moderate risk of bias

Abbreviations: RSV—respiratory syncytial virus; RCT—randomized controlled trial; GA—gestational age; BPD—bronchopulmonary dysplasia; CHD—congenital heart defects; AEs—adverse events; CLD—chronic lung disease.

**Table 4:** Summary of nirsevimab safety.

Study (Author, Year)	Design/Sample Size	Population	Intervention	Key Findings	Newcastle-Ottawa Scale/Cochrane RoB
Domachowske et al., 2022 (MEDLEY) [54]	RCT/N = 918 (612 preterm (406 nirsevimab/206 palivizumab), 306 CHD–CLD (208 nirsevimab/98 palivuzumab)	Infants with CHD and/or CLD of prematurity entering the first RSV season	Nirsevimab single dose vs. palivizumab monthly × 5 doses	Similar safety/tolerability; 5 deaths in the nirsevimab group not attributed to the drug.	Low risk of bias
Hammitt et al., 2022 (MELODY) [32]	RCT/N = 1490 (994 nirsevimab; 496 placebo)	Healthy late-preterm and term infants entering the first RSV season	Nirsevimab single dose (50 mg <5 kg; 100 mg ≥5 kg) vs. placebo	Serious adverse events occurred in 6.8% of the nirsevimab group and 7.3% of the placebo group; 3 deaths occurred in the nirsevimab group, all considered unrelated to the study drug by investigators.	Low risk of bias
Drysdale et al., 2023 (HARMONIE) [41]	Pragmatic RCT/N = 8058 (4037 nirsevimab; 4021 no RSV prophylaxis)	Healthy infants ≥ 29 weeks’ gestation entering their first RSV season	Nirsevimab single dose vs. standard care (no RSV prophylaxis)	Favorable safety profile, 2.1% treatment-related adverse events, no serious adverse events or hypersensitivity reactions related to nirsevimab.	Low risk of bias
Mallah et al., 2024 [55]	Population-based longitudinal observational/N = 9408	Infants in Galicia, Spain	nirsevimab immunization	No severe adverse events related to nirsevimab were registered in real-world use.	Moderate risk of bias
Ernst et al., 2024 [56]	Population-based observational study/N = 1277	Infants from Luxembourg	Universal nirsevimab immunization	No adverse events related to nirsevimab were reported.	Moderate risk of bias
Consolati et al., 2024 [58]	Prospective observational cohort/N = 292	Newborns born in Valle d’Aosta, Italy	Universal nirsevimab prophylaxis	A few mild transient side effects were reported.	Moderate risk of bias
Carcione et al., 2025 [57]	Active post-marketing safety surveillance/N = 1195	Children receiving nirsevimab in Western Australia (April–July 2024)	Universal nirsevimab immunization	No serious adverse events attributable to nirsevimab were identified; reported adverse events were predominantly mild and transient (local injection-site reactions, fever, irritability); no cases of anaphylaxis or safety signals were detected, supporting a favorable real-world safety profile.	Moderate risk of bias

Abbreviations: RSV—respiratory syncytial virus; RCT—randomized controlled trial; CHD—congenital heart defects; CLD—chronic lung disease.

**Table 5:** Summary of palivizumab cost-effectiveness.

Study (Author, Year)	Design	Population	Intervention	Key Findings	Newcastle-Ottawa Scale/Cochrane RoB
El Hassan et al., 2006 [59]	Decision-analytic cost-effectiveness model	Premature infants (<33 weeks) without CLD (USA)	Palivizumab prophylaxis vs. no prophylaxis	Palivizumab was not cost-effective across gestational ages; ICERs ranged from ~$675,780 to over $1,850,000 per QALY gained; prophylaxis costs outweighed savings even when assuming asthma risk benefit.	Moderate risk of bias
Lanctôt et al., 2008 [60]	Decision-analytic cost-effectiveness model	Infants born at 32–35 weeks’ GA without CLD (Canada)	Palivizumab prophylaxis vs. no prophylaxis	Palivizumab was cost-effective in preventing RSV hospitalizations in infants 32–35 weeks’ gestation, with favorable ICERs in many modeled scenarios; cost-effectiveness was driven by hospitalization reduction and high hospital costs.	Moderate risk of bias
Tam et al., 2009 [61]	Decision-analytic cost-effectiveness model	Term Inuit infants in the Eastern Canadian Arctic	Palivizumab prophylaxis vs. no prophylaxis	Palivizumab prophylaxis was cost-effective in Inuit infants at high risk of RSV, with ICERs below commonly accepted thresholds for cost-effectiveness based on local epidemiology and healthcare costs.	Moderate risk of bias
Nuijten and Wittenberg, 2010 [62]	Decision-analytic cost-effectiveness model	Premature infants 32–35 wGA with ≥2 risk factors (Spain)	Palivizumab prophylaxis vs. no prophylaxis	Palivizumab was cost-effective; ICER 6142–12,814 €/QALY, below the Spanish WTP threshold (~30,000 €/QALY).	Moderate risk of bias
Resch et al., 2012 [63]	Long-term epidemiologic cost-effectiveness analysis	High-risk infants (Austria)	Palivizumab prophylaxis vs. no prophylaxis	Palivizumab was cost-effective in high-risk infants; ICER per QALY ranged from €8484 to €26,292 depending on the subgroup.	Moderate risk of bias
Narayan et al., 2020 [64]	Decision-tree cost-effectiveness analysis	High-risk infants (CHD, BPD, preterm) in the UK	Palivizumab prophylaxis vs. no prophylaxis	Palivizumab was cost-effective in preventing severe RSV hospitalizations in a broader UK population than current guidelines, including being dominant (cost saving + effective) in several subgroups.	Moderate risk of bias
Rodgers-Gray et al., 2023 [65]	Decision-tree cost-utility model	Moderate-to-late preterm infants (32–35 weeks’ GA, Canada)	Palivizumab prophylaxis vs. no prophylaxis	Cost per QALY was $29,789 using IRST (79% probability < $50,000/QALY) and $15,833 using CRST (96% probability < $50,000/QALY); cost-effectiveness improved with vial sharing.	Moderate risk of bias

Abbreviations: RSV—respiratory syncytial virus; GA—gestational age; BPD—bronchopulmonary dysplasia; CHD—congenital heart defects; CLD—chronic lung disease; ICERs—incremental cost-effectiveness ratios; QALY—quality-adjusted life year.

**Table 6:** Summary of nirsevimab cost-effectiveness.

Study (Author, Year)	Design	Population	Intervention	Key Findings	Newcastle-Ottawa Scale/Cochrane RoB
Hodgson et al., 2022 [67]	Dynamic transmission cost-effectiveness model	All infants (England and Wales)	Universal nirsevimab prophylaxis vs. palivizumab program/no prophylaxis	Nirsevimab could be cost-effective if priced ≤ £63 per dose (seasonal) or ≤ £32 (season + catch-up) at a £20,000/QALY threshold;	Moderate risk of bias
Hutton et al., 2024 [66]	Decision-analytic cost-effectiveness model	Infants <8 months entering the first RSV season (USA)	Nirsevimab prophylaxis vs. no prophylaxis	Base case ICER ~USD 153,517/QALY gained; cost-effectiveness sensitive to drug price, RSV hospitalization costs, and quality-of-life losses; some scenarios showed nirsevimab cost-saving or ICER < 100,000 USD/QALY under favorable assumptions	Moderate risk of bias
Bugden et al., 2025 [68]	Decision-analytic cost-effectiveness model	Canadian infants <1 yr (regional risk strata)	Various nirsevimab strategies vs. palivizumab/no intervention	Replacement of palivizumab with nirsevimab is cost-saving and more effective nationwide; optimal expanded coverage depends on dose price and regional RSV risk (e.g., savings up to $1067.03 and QALY gains 0.000884 per infant in Nunavut); universal nirsevimab is cost-effective at threshold prices below ~$112/dose.	Moderate risk of bias
Gil-Prieto et al., 2025 [69]	Cost–utility modeling study	Neonates and infants (Spain)	Nirsevimab prophylaxis vs. the standard of practice	Nirsevimab could be cost-effective in preventing RSV at an acquisition price justifiable under WTP thresholds; ICERs and economically justifiable prices depend on dose cost (e.g., €220 base), with savings from averted hospitalizations and RSV cases.	Moderate risk of bias
Bini et al., 2025 [70]	Cost–utility modeling study)	Neonates and infants (Italy)	Nirsevimab prophylaxis vs. the standard of practice	Economically justifiable price ≈ €267–€400 per QALY for all infants; indirect cost inclusion increases economically justifiable price values; supports universal immunization cost-effectiveness	Moderate risk of bias
Zeevat et al., 2025 [71]	Static cost-effectiveness model	All infants entering the first RSV season (Netherlands)	Universal infant nirsevimab vs. standard care (palivizumab for high risk)	Universal nirsevimab could be cost-effective with an economically justifiable acquisition price of ~€220/dose at a willingness-to-pay threshold of €50,000/QALY; prevented thousands of cases and hospitalizations compared with the standard of care	Moderate risk of bias
Noto et al., 2025 [72]	Static decision analytic model	All infants entering the first RSV season (Japan)	Nirsevimab universal prophylaxis vs. SoP (palivizumab for high-risk)	ICER ≈ ¥4,537,256/QALY (~cost-effective at Japanese WTP ¥5,000,000/QALY); societal perspective ICER ≈ ¥1,695,635/QALY.	Moderate risk of bias
Wang et al., 2025 [73]	Markov decision-tree cost-effectiveness model	Infants in Shanghai, China	Nirsevimab immunization (seasonal vs. year-round) vs. no intervention	Both seasonal and year-round nirsevimab strategies were cost-effective compared with no intervention at the willingness-to-pay threshold set at GDP per capita; the seasonal approach yielded lower ICERs than year-round administration.	Moderate risk of bias

Abbreviations: RSV—respiratory syncytial virus; ICERs—incremental cost-effectiveness ratios; QALY—quality-adjusted life year; GDP—gross domestic product. Overall, cost-effectiveness estimates varied substantially across settings and were highly sensitive to acquisition price, healthcare costs, and willingness-to-pay thresholds, highlighting the context-specific nature of economic evaluations.

**Table 7:** Country-specific cost-effectiveness thresholds for nirsevimab.

Study (Author, Year)	Country	WTP Threshold	Cost-Effective Price/ICER Summary
Hodgson et al., 2022 [67]	England and Wales	£20,000/QALY	Universal program is cost-effective only at a lower acquisition price (if the PPPD is ≤£63)
Hutton et al., 2024 [66]	USA	Not explicitly defined	US $153,517 (first season)–$308 468 (second season) QALY; Potentially cost-effective for all the infants in the first season and for those with higher risk in the second season.
Bugden et al., 2025 [68]	Canada	Canadian $100,000/QALY	Price and region dependent; cost-effective at ≤Canadian $306 PPD in southern Canada and at ≤$685 PPD in northwest Canada
Gil-Prieto et al., 2025 [69]	Spain	€0–€30,000/QALY	Estimated economically justifiable PPD €222–€415
Bini et al., 2025 [70]	Italy	€0/QALY, €22,000/QALY and €30,000/QALY	Cost-effective at PPD ≈ €267–€400
Zeevat et al., 2025 [71]	Netherlands	€50,000/QALY	Cost-effective if ~€220/dose
Noto et al., 2025 [72]	Japan	¥5,000,000/QALY	¥1,695,635–4,537,256/QALY ((cost-effective)
Wang et al., 2025 [73]	China	GDP per capita (US$26,866)	US$732,413 per QALY at the seasonal approach (newborns born in October-February)

Abbreviations: QALY—quality-adjusted life year; WTP—willingness to pay; PPD—price per dose; GDP—gross domestic product.

## Data Availability

No new data were created or analyzed in this study.

## Abbreviations

The following abbreviations are used in this manuscript:

BPD	Bronchopulmonary dysplasia
CHD	Congenital heart defects
CLD	Chronic lung disease
CRST	Canadian Risk Scoring Tool
EMA	European Medicines Agency
FDA	Food and Drug Administration
ICERs	Incremental cost-effectiveness ratios
ICU	Intensive care unit
IRST	International Risk Scoring Tool
LRTI	Lower respiratory tract infections
nAb	Neutralizing antibody
QALY	Cost per quality-adjusted life year
RCTs	randomized controlled trials
RSV	Respiratory syncytial virus

## References

[1] Zar H.J., Cacho F., Kootbodien T., Mejias A., Ortiz J.R., Stein R.T., Hartert T. (2024). V Early-Life Respiratory Syncytial Virus Disease and Long-Term Respiratory Health. Lancet Respir. Med..

[2] Li Y., Wang X., Blau D.M., Caballero M.T., Feikin D.R., Gill C.J., Madhi S.A., Omer S.B., Simões E.A.F., Campbell H. (2022). Global, Regional, and National Disease Burden Estimates of Acute Lower Respiratory Infections Due to Respiratory Syncytial Virus in Children Younger than 5 Years in 2019: A Systematic Analysis. Lancet.

[3] Shi T., McAllister D.A., O’Brien K.L., Simoes E.A.F., Madhi S.A., Gessner B.D., Polack F.P., Balsells E., Acacio S., Aguayo C. (2017). Global, Regional, and National Disease Burden Estimates of Acute Lower Respiratory Infections Due to Respiratory Syncytial Virus in Young Children in 2015: A Systematic Review and Modelling Study. Lancet.

[4] Abu-Raya B., Viñeta Paramo M., Reicherz F., Lavoie P.M. (2023). Why Has the Epidemiology of RSV Changed during the COVID-19 Pandemic?. eClinicalMedicine.

[5] Issa M., Lagare A., Bachir G.A.M., Bowo-Ngandji A., Hassane F., Magagi L.H., Mahamadou D., Seini H., Adehossi E., Zoubeirou A.M. (2025). Respiratory Syncytial Virus Epidemiology During and After Covid-19 Pandemic in Africa: Systematic Review and Meta-Analysis. Health Sci. Rep..

[6] Trigueros Montes J.B., Montes D., Miele A., Baik-Han W., Gulati G., Lew L.Q. (2024). The Impact of COVID-19 Pandemic on Respiratory Syncytial Virus Infection in Children. Pulm. Med..

[7] Drăgănescu A.C., Miron V.D., Săndulescu O., Bilaşco A., Streinu-Cercel A., Sandu R.G., Marinescu A., Gunșahin D., Hoffmann K.I., Horobeț D.Ș. (2023). Omicron in Infants—Respiratory or Digestive Disease?. Diagnostics.

[8] Pavelescu M.L., Dinulescu A., Păsărică A.-S., Dijmărescu I., Păcurar D. (2024). Hematological Profile, Inflammatory Markers and Serum Liver Enzymes in COVID 19 Positive Children vs. COVID 19 Negative Ones—A Comparative Study. Front. Pediatr..

[9] Hall C.B., Weinberg G.A., Iwane M.K., Blumkin A.K., Edwards K.M., Staat M.A., Auinger P., Griffin M.R., Poehling K.A., Erdman D. (2009). The Burden of Respiratory Syncytial Virus Infection in Young Children. N. Engl. J. Med..

[10] Tenenbaum T., Liese J., Welte T., Rademacher J. (2024). Respiratory Syncytial Virus-Associated Respiratory Diseases in Children and Adults. Dtsch. Arztebl. Int..

[11] Korten I., Kieninger E., Klenja S., Mack I., Schläpfer N., Barbani M.T., Regamey N., Kuehni C.E., Hilty M., Frey U. (2018). Respiratory Viruses in Healthy Infants and Infants with Cystic Fibrosis: A Prospective Cohort Study. Thorax.

[12] Poamaneagra S.C., Plesca D.-A., Tataranu E., Marginean O., Nemtoi A., Mihai C., Gilca-Blanariu G.-E., Andronic C.-M., Anchidin-Norocel L., Diaconescu S. (2024). A Global Perspective on Transition Models for Pediatric to Adult Cystic Fibrosis Care: What Has Been Made So Far?. J. Clin. Med..

[13] Georgescu G., Chemaly R.F. (2009). Palivizumab: Where to from Here?. Expert Opin. Biol. Ther..

[14] Garegnani L., Roson Rodriguez P., Escobar Liquitay C.M., Esteban I., Franco J.V. (2025). Palivizumab for Preventing Severe Respiratory Syncytial Virus (RSV) Infection in Children. Cochrane Database Syst. Rev..

[15] Null D., Bimle C., Weisman L., Johnson K., Steichen J., Singh S., Wang E., Asztalos E., Loeffler A., Azimi P. (1998). The IMpact-RSV Study Group Palivizumab, a Humanized Respiratory Syncytial Virus Monoclonal Antibody, Reduces Hospitalization from Respiratory Syncytial Virus Infection in High-Risk Infants. The IMpact-RSV Study Group. Pediatrics.

[16] Lanari M., Vandini S., Arcuri S., Galletti S., Faldella G. (2013). The Use of Humanized Monoclonal Antibodies for the Prevention of Respiratory Syncytial Virus Infection. Clin. Dev. Immunol..

[17] Mori M., Yoshizaki K., Watabe S., Ishige M., Hinoki A., Kondo T., Taguchi T., Hasegawa H., Hatata T., Tanuma N. (2023). Safety, Efficacy and Pharmacokinetics of Palivizumab in off-Label Neonates, Infants, and Young Children at Risk for Serious Respiratory Syncytial Virus Infection: A Multicenter Phase II Clinical Trial. Lancet Reg. Health West. Pac..

[18] Brady M.T., Byington C.L., Davies H.D., Edwards K.M., Jackson M.A., Maldonado Y.A., Murray D.L., Orenstein W.A., Rathore M.H., Sawyer M.H. (2014). Updated Guidance for Palivizumab Prophylaxis Among Infants and Young Children at Increased Risk of Hospitalization for Respiratory Syncytial Virus Infection. Pediatrics.

[19] Anderson E.J., Carosone-Link P., Yogev R., Yi J., Simões E.A.F. (2017). Effectiveness of Palivizumab in High-Risk Infants and Children. Pediatr. Infect. Dis. J..

[20] Balbi H. (2024). Nirsevimab: A Review. Pediatr. Allergy Immunol. Pulmonol..

[21] Griffin M.P., Yuan Y., Takas T., Domachowske J.B., Madhi S.A., Manzoni P., Simões E.A.F., Esser M.T., Khan A.A., Dubovsky F. (2020). Single-Dose Nirsevimab for Prevention of RSV in Preterm Infants. N. Engl. J. Med..

[22] Keam S.J. (2023). Nirsevimab: First Approval. Drugs.

[23] Nazir A., Fatima R., Nazir A. (2023). FDA Grants Approval to the RSV Vaccine (Nirsevimab-Alip) for All Infants: A Leap Forward for Shielding the Smallest. Int. J. Surg..

[24] Robbie G.J., Criste R., Dall’Acqua W.F., Jensen K., Patel N.K., Losonsky G.A., Griffin M.P. (2013). A Novel Investigational Fc-Modified Humanized Monoclonal Antibody, Motavizumab-YTE, Has an Extended Half-Life in Healthy Adults. Antimicrob. Agents Chemother..

[25] Sumsuzzman D.M., Wang Z., Langley J.M., Moghadas S.M. (2025). Real-World Effectiveness of Nirsevimab against Respiratory Syncytial Virus Disease in Infants: A Systematic Review and Meta-Analysis. Lancet Child Adolesc. Health.

[26] Tanashat M., Abuelazm M., Manasrah A., Altobaishat O., Masadeh N.M., Abouzid M. (2025). Efficacy and Safety of Nirsevimab for Preventing Respiratory Syncytial Virus Infection in Infants: An Updated Systematic Review and Meta-Analysis Encompassing 11,001 Participants. Bayl. Univ. Med. Cent. Proc..

[27] Abu-Raya B., Langley J.M., Lavoie P. (2024). Nirsevimab to Reduce Infant Morbidity from Respiratory Syncytial Virus. Can. Med. Assoc. J..

[28] Feltes T.F., Cabalka A.K., Meissner H.C., Piazza F.M., Carlin D.A., Top F.H., Connor E.M., Sondheimer H.M., Cardiac Synagis Study Group (2003). Palivizumab Prophylaxis Reduces Hospitalization Due to Respiratory Syncytial Virus in Young Children with Hemodynamically Significant Congenital Heart Disease. J. Pediatr..

[29] Frogel M., Nerwen C., Cohen A., VanVeldhuisen P., Harrington M., Boron M. (2008). Prevention of Hospitalization Due to Respiratory Syncytial Virus: Results from the Palivizumab Outcomes Registry. J. Perinatol..

[30] Yeo K.T., Yung C.F., Khoo P.C., Saffari S.E., Sng J.S.P., How M.S., Quek B.H. (2021). Effectiveness of Palivizumab Against Respiratory Syncytial Virus Hospitalization Among Preterm Infants in a Setting With Year-Round Circulation. J. Infect. Dis..

[31] Manzoni P., Baraldi E., Luna M.S., Tzialla C. (2022). Real-World Studies of Respiratory Syncytial Virus Hospitalizations among Moderate/Late Preterm Infants Exposed to Passive Immunoprophylaxis with Palivizumab. Am. J. Perinatol..

[32] Hammitt L.L., Dagan R., Yuan Y., Baca Cots M., Bosheva M., Madhi S.A., Muller W.J., Zar H.J., Brooks D., Grenham A. (2022). Nirsevimab for Prevention of RSV in Healthy Late-Preterm and Term Infants. N. Engl. J. Med..

[33] Pelletier J.H., Rush S.Z., Robinette E., Maholtz D.E., Bigham M.T., Forbes M.L., Shein S.L., Karsies T.J., Horvat C.M. (2025). Nirsevimab Administration and RSV Hospitalization in the 2024–2025 Season. JAMA Netw. Open.

[34] Rodríguez-Fernández R., González-Martínez F., Ojeda Velázquez I., Rodríguez Díaz M., Capozzi Bucciol M.V., González-Sánchez M.I., Pérez-Moreno J., Toledo del Castillo B. (2024). Nirsevimab Effectiveness against Hospital Admission for Respiratory Syncytial Virus Bronchiolitis in Infants. Rev. Española Quimioter..

[35] Ezpeleta G., Navascués A., Viguria N., Herranz-Aguirre M., Juan Belloc S.E., Gimeno Ballester J., Muruzábal J.C., García-Cenoz M., Trobajo-Sanmartín C., Echeverria A. (2024). Effectiveness of Nirsevimab Immunoprophylaxis Administered at Birth to Prevent Infant Hospitalisation for Respiratory Syncytial Virus Infection: A Population-Based Cohort Study. Vaccines.

[36] Costantino C., Amodio E., Asciutto R., Affranchi C., Belbruno F., Bonaccorso N., Cilia S., Contarino F.M., Di Gaetano V., Di Gregorio F. (2025). Impact of a Universal Nirsevimab Prevention Program Against Respiratory Syncytial Virus Bronchiolitis in Infants in Sicily (Italy) During the 2024–2025 Epidemic Season: A Retrospective Cohort Study. Vaccines.

[37] Höck M., Borena W., Brunner J., Wechselberger K., Scheiring J., Ralser E., Pupp Peglow U., Wöckinger P., D’Costa E., Kaiser V. (2025). Acceptance and Impact of Nirsevimab and the RSVpreF Vaccine Following Implementation in Austria. Front. Public Health.

[38] Zambrano L.D., Simeone R.M., Newhams M.M., Payne A.B., Halasa N.B., Orzel-Lockwood A.O., Calixte J.M., Kamidani S., Crandall H., Cameron M.A. (2025). Nirsevimab Effectiveness Against Intensive Care Unit Admission for Respiratory Syncytial Virus in Infants—24 States, December 2024–April 2025. MMWR Morb. Mortal. Wkly. Rep..

[39] Xu H., Aparicio C., Wats A., Araujo B.L., Pitzer V.E., Warren J.L., Shapiro E.D., Niccolai L.M., Weinberger D.M., Oliveira C.R. (2025). Estimated Effectiveness of Nirsevimab Against Respiratory Syncytial Virus. JAMA Netw. Open.

[40] Villa S., Scarioni S., Pigozzi E., Maffeo M., Maistrello M., Bagarella G., Scovenna F., Morani F., Romano M., Zuccotti G. (2026). Reduced Emergency Department Visits and Hospitalizations in Infants after Universal Respiratory Syncytial Virus Immunization, Italy, 2024–25. Emerg. Infect. Dis..

[41] Drysdale S.B., Cathie K., Flamein F., Knuf M., Collins A.M., Hill H.C., Kaiser F., Cohen R., Pinquier D., Felter C.T. (2023). Nirsevimab for Prevention of Hospitalizations Due to RSV in Infants. N. Engl. J. Med..

[42] Moline H.L., Tannis A., Toepfer A.P., Williams J.V., Boom J.A., Englund J.A., Halasa N.B., Staat M.A., Weinberg G.A., Selvarangan R. (2024). Early Estimate of Nirsevimab Effectiveness for Prevention of Respiratory Syncytial Virus–Associated Hospitalization Among Infants Entering Their First Respiratory Syncytial Virus Season—New Vaccine Surveillance Network, October 2023–February 2024. MMWR Morb. Mortal. Wkly. Rep..

[43] Soudani S., Bertizzolo L., Ghemmouri M., Chappell M., McCool R., Reddish K., Miller P., Barker E., Fewster H. (2025). Nirsevimab for Preventing Respiratory Syncytial Virus Lower Respiratory Tract Infections in Infants: A Systematic Review and Meta-Analysis. Front. Public Health.

[44] Carbonell-Estrany X., Simões E.A.F., Bont L., Manzoni P., Zar H.J., Greenough A., Ramilo O., Stein R., Law B., Mejias A. (2025). Twenty-Five Years of Palivizumab: A Global Historic Review of Its Impact on the Burden of Respiratory Syncytial Virus Disease in Children. Expert Rev. Anti. Infect. Ther..

[45] Siva Subramanian K.N., Weisman L.E., Rhodes T., Ariagno R., Sánchez P.J., Steichen J., Givner L.B., Jennings T.L., Top F.H., Carlin D. (1998). Safety, Tolerance and Pharmacokinetics of a Humanized Monoclonal Antibody to Respiratory Syncytial Virus in Premature Infants and Infants with Bronchopulmonary Dysplasia. Pediatr. Infect. Dis. J..

[46] O’Hagan S., Galway N., Shields M., Mallett P., Groves H. (2023). Review of the Safety, Efficacy and Tolerability of Palivizumab in the Prevention of Severe Respiratory Syncytial Virus (RSV) Disease. Drug Healthc. Patient Saf..

[47] Meissner H.C., Groothuis J.R., Rodriguez W.J., Welliver R.C., Hogg G., Gray P.H., Loh R., Simoes E.A.F., Sly P., Miller A.K. (1999). Safety and Pharmacokinetics of an Intramuscular Monoclonal Antibody (SB 209763) against Respiratory Syncytial Virus (RSV) in Infants and Young Children at Risk for Severe RSV Disease. Antimicrob. Agents Chemother..

[48] Groothuis J.R. (2003). Safety of Palivizumab in Preterm Infants 29 to 32 Weeks’ Gestational Age Without Chronic Lung Disease to Prevent Serious Respiratory Syncytial Virus Infection. Eur. J. Clin. Microbiol. Infect. Dis..

[49] Null D., Pollara B., Dennehy P.H., Steichen J., S??nchez P.J., Givner L.B., Carlin D., Landry B., Top F.H., Connor E. (2005). Safety and immunogenicity of palivizumab (SYNAGIS) administered for two seasons. Pediatr. Infect. Dis. J..

[50] Groothuis J.R. (2001). Safety and tolerance of palivizumab administration in a large northern hemisphere trial. Pediatr. Infect. Dis. J..

[51] Lacaze-Masmonteil T., Seidenberg J., Mitchell I., Cossey V., Cihar M., Csader M., Baarsma R., Valido M., Pollack P.F., Groothuis J.R. (2003). Evaluation of the Safety of Palivizumab in the Second Season of Exposure in Young Children at Risk for Severe Respiratory Syncytial Virus Infection. Drug Saf..

[52] Kashiwagi T., Okada Y., Nomoto K. (2018). Palivizumab Prophylaxis Against Respiratory Syncytial Virus Infection in Children with Immunocompromised Conditions or Down Syndrome: A Multicenter, Post-Marketing Surveillance in Japan. Pediatr. Drugs.

[53] Castillo L.M., Bugarin G., Arias J.C., Barajas Rangel J.I., Serra M.E., Vain N. (2017). One-Year Observational Study of Palivizumab Prophylaxis on Infants at Risk for Respiratory Syncytial Virus Infection in Latin America. J. Pediatr..

[54] Domachowske J., Madhi S.A., Simões E.A.F., Atanasova V., Cabañas F., Furuno K., Garcia-Garcia M.L., Grantina I., Nguyen K.A., Brooks D. (2022). Safety of Nirsevimab for RSV in Infants with Heart or Lung Disease or Prematurity. N. Engl. J. Med..

[55] Mallah N., Pardo-Seco J., Pérez-Martínez O., Durán-Parrondo C., Martinón-Torres F., Mallah N., Pardo-Seco J., Santiago-Pérez M.-I., Pérez-Martínez O., Otero-Barrós M.-T. (2025). Full 2023–24 Season Results of Universal Prophylaxis with Nirsevimab in Galicia, Spain: The NIRSE-GAL Study. Lancet Infect. Dis..

[56] Ernst C., Bejko D., Gaasch L., Hannelas E., Kahn I., Pierron C., Del Lero N., Schalbar C., Do Carmo E., Kohnen M. (2024). Impact of Nirsevimab Prophylaxis on Paediatric Respiratory Syncytial Virus (RSV)-Related Hospitalisations during the Initial 2023/24 Season in Luxembourg. Eurosurveillance.

[57] Carcione D., Spencer P., Pettigrew G., Leeb A., Drake-Brockman C., Ford T., Effler P. (2025). Active Post-Marketing Safety Surveillance of Nirsevimab Administered to Children in Western Australia, April–July 2024. Pediatr. Infect. Dis. J..

[58] Consolati A., Farinelli M., Serravalle P., Rollandin C., Apprato L., Esposito S., Bongiorno S. (2024). Safety and Efficacy of Nirsevimab in a Universal Prevention Program of Respiratory Syncytial Virus Bronchiolitis in Newborns and Infants in the First Year of Life in the Valle d’Aosta Region, Italy, in the 2023–2024 Epidemic Season. Vaccines.

[59] ElHassan N.O., Sorbero M.E.S., Hall C.B., Stevens T.P., Dick A.W. (2006). Cost-Effectiveness Analysis of Palivizumab in Premature Infants Without Chronic Lung Disease. Arch. Pediatr. Adolesc. Med..

[60] Lanctôt K.L., Masoud S.T., Paes B.A., Tarride J.-E., Chiu A., Hui C., Francis P.L., Oh P.I. (2008). The Cost-Effectiveness of Palivizumab for Respiratory Syncytial Virus Prophylaxis in Premature Infants with a Gestational Age of 32–35 Weeks: A Canadian-Based Analysis. Curr. Med. Res. Opin..

[61] Tam D.Y., Banerji A., Paes B.A., Hui C., Tarride J.-E., Lanctôt K.L. (2009). The Cost Effectiveness of Palivizumab in Term Inuit Infants in the Eastern Canadian Arctic. J. Med. Econ..

[62] Nuijten M.J., Wittenberg W. (2010). Cost Effectiveness of Palivizumab in Spain: An Analysis Using Observational Data. Eur. J. Health Econ..

[63] Resch B., Sommer C., Nuijten M.J.C., Seidinger S., Walter E., Schoellbauer V., Mueller W.D. (2012). Cost-Effectiveness of Palivizumab for Respiratory Syncytial Virus Infection in High-Risk Children, Based on Long-Term Epidemiologic Data From Austria. Pediatr. Infect. Dis. J..

[64] Narayan O., Bentley A., Mowbray K., Hermansson M., Pivonka D., Kemadjou E.N., Belsey J. (2020). Updated Cost-Effectiveness Analysis of Palivizumab (Synagis) for the Prophylaxis of Respiratory Syncytial Virus in Infant Populations in the UK. J. Med. Econ..

[65] Rodgers-Gray B.S., Fullarton J.R., Carbonell-Estrany X., Keary I.P., Tarride J.-É., Paes B.A. (2023). Impact of Using the International Risk Scoring Tool on the Cost-Utility of Palivizumab for Preventing Severe Respiratory Syncytial Virus Infection in Canadian Moderate-to-Late Preterm Infants. J. Med. Econ..

[66] Hutton D.W., Prosser L.A., Rose A.M., Mercon K., Ortega-Sanchez I.R., Leidner A.J., McMorrow M.L., Fleming-Dutra K.E., Prill M.M., Pike J. (2024). Cost-Effectiveness of Nirsevimab for Respiratory Syncytial Virus in Infants and Young Children. Pediatrics.

[67] Hodgson D., Koltai M., Krauer F., Flasche S., Jit M., Atkins K.E. (2022). Optimal Respiratory Syncytial Virus Intervention Programmes Using Nirsevimab in England and Wales. Vaccine.

[68] Bugden S., Mital S., Nguyen H.V. (2025). Cost-Effectiveness of Nirsevimab and Maternal RSVpreF for Preventing Respiratory Syncytial Virus Disease in Infants across Canada. BMC Med..

[69] Gil-Prieto R., Pérez-Martín J., Díaz Aguiló A., Soudani S., Platero-Alonso L., López-Belmonte J.L., de la Cuadra-Grande A., Casado M.Á., Álvarez Aldean J. (2025). Estimating the Economically Justifiable Price of Nirsevimab versus Standard of Practice for the Prevention of Respiratory Syncytial Virus Infections in Neonates and Infants in Spain: A Cost–Utility Modelling Study. BMJ Public Health.

[70] Bini C., Marcellusi A., Cazzato D., Muzii B., Soudani S., Bozzola E., Midulla F., Baraldi E., Bonanni P., Boccalini S. (2025). Cost-Effectiveness Analysis of Nirsevimab for the Prevention of Respiratory Syncytial Virus among Italian Infants. Clin. Drug Investig..

[71] Zeevat F., van der Pol S., Kieffer A., Postma M.J., Boersma C. (2025). Cost-Effectiveness Analysis of Nirsevimab for Preventing Respiratory Syncytial Virus-Related Lower Respiratory Tract Disease in Dutch Infants: An Analysis Including All-Infant Protection. Pharmacoeconomics.

[72] Noto S., Kieffer A., Soudani S., Arashiro T., Tadera C., Eymere S., Lemański T., Wang X. (2025). Cost-Effectiveness and Public Health Impact of Universal Prophylaxis with Nirsevimab Against Respiratory Syncytial Virus (RSV) Infections in All Infants in Japan. Infect. Dis. Ther..

[73] Wang Q., Wu J., Li L., Guo Z., Zheng B., Zhang S., Xiang C., Li M., Qiao X., Lv X. (2025). Cost-Effectiveness Analysis of Nirsevimab for Prevention of Respiratory Syncytial Virus Disease among Infants in Shanghai, China: A Modeling Study. Hum. Vaccin. Immunother..

[74] Reicherz F., Abu-Raya B., Akinseye O., Rassekh S.R., Wiens M.O., Lavoie P.M. (2024). Efficacy of Palivizumab Immunoprophylaxis for Reducing Severe RSV Outcomes in Children with Immunodeficiencies: A Systematic Review. J. Pediatr. Infect. Dis. Soc..

[75] El-Atawi K., De Luca D., Ramanathan R., Sanchez Luna M., Alsaedi S., Abdul Wahab M.G., Hamdi M., Saleh M. (2023). Efficacy and Safety of Palivizumab as a Prophylaxis for Respiratory Syncytial Virus (RSV) Disease: An Updated Systemic Review and Meta-Analysis. Cureus.

[76] Resch B. (2014). Respiratory Syncytial Virus Infection in High-Risk Infants—An Update on Palivizumab Prophylaxis. Open Microbiol. J..

[77] Trusinska D., Lee B., Ferdous S., Kwok H.H.Y., Gordon B., Gao J., Ma L., Xiong H., Sheikh S.A., Schwarze J. (2025). Real-World Uptake of Nirsevimab, RSV Maternal Vaccine, and RSV Vaccines for Older Adults: A Systematic Review and Meta-Analysis. EClinicalMedicine.

[78] Wang X., Kong L., Liu X., Wu P., Zhang L., Ding F. (2025). Effectiveness of Nirsevimab Immunization against RSV Infection in Preterm Infants: A Systematic Review and Meta-Analysis. Front. Immunol..

[79] Lien H.-C., Lien C.-H., Liu T.-Y., Weng S.-L., Tai Y.-L., Huang Y.-N., Chi H., Chiu N.-C., Yeung C.-Y., Lin C.-Y. (2025). Efficacy of Nirsevimab for the Prevention of RSV Disease in Infants: A Systematic Review, Meta-Analysis of Randomized Controlled Trials, and Global Perspectives on Recommendations and Unmet Needs. Pediatr. Neonatol..

[80] Wang D., Bayliss S., Meads C. (2011). Palivizumab for Immunoprophylaxis of Respiratory Syncitial Virus (RSV) Bronchiolitis in High-Risk Infants and Young Children: A Systematic Review and Additional Economic Modelling of Subgroup Analyses. Health Technol. Assess..

[81] Hussman J.M., Li A., Paes B., Lanctôt K.L. (2012). A Review of Cost–Effectiveness of Palivizumab for Respiratory Syncytial Virus. Expert Rev. Pharmacoecon. Outcomes Res..

[82] Wang D., Cummins C., Bayliss S., Sandercock J., Burls A. (2008). Immunoprophylaxis against Respiratory Syncytial Virus (RSV) with Palivizumab in Children: A Systematic Review and Economic Evaluation. Health Technol. Assess..

[83] Mac S., Sumner A., Duchesne-Belanger S., Stirling R., Tunis M., Sander B. (2019). Cost-Effectiveness of Palivizumab for Respiratory Syncytial Virus: A Systematic Review. Pediatrics.

[84] Nuijten M., Lebmeier M., Wittenberg W. (2009). Cost Effectiveness of Palivizumab in Children with Congenital Heart Disease in Germany. J. Med. Econ..

[85] Killikelly A., Siu W., Brousseau N. (2025). Summary of the National Advisory Committee on Immunization (NACI) Statement on the Prevention of Respiratory Syncytial Virus (RSV) in Infants. Can. Commun. Dis. Rep..

[86] Shoukat A., Abdollahi E., Galvani A.P., Halperin S.A., Langley J.M., Moghadas S.M. (2023). Cost-Effectiveness Analysis of Nirsevimab and Maternal RSVpreF Vaccine Strategies for Prevention of Respiratory Syncytial Virus Disease among Infants in Canada: A Simulation Study. Lancet Reg. Health Am..

[87] Kieffer A., Sardesai A., Musci R., Beuvelet M., Tribaldos Causadias de Su M., Lee J.K.H., Rizzo C., Greenberg M. (2023). EE438 Cost-Effectiveness of Nirsevimab Against Respiratory Syncytial Virus Lower Respiratory Tract Disease (RSV LRTD) in the US Birth Cohort. Value Health.

[88] Kampmann B., Madhi S.A., Munjal I., Simões E.A.F., Pahud B.A., Llapur C., Baker J., Pérez Marc G., Radley D., Shittu E. (2023). Bivalent Prefusion F Vaccine in Pregnancy to Prevent RSV Illness in Infants. N. Engl. J. Med..

[89] Santilli V., Sgrulletti M., Costagliola G., Beni A., Mastrototaro M.F., Montin D., Rizzo C., Martire B., Miraglia del Giudice M., Moschese V. (2025). Maternal Immunization: Current Evidence, Progress, and Challenges. Vaccines.

[90] Cirillo A.A., Zeme M., Morales A., Rahseparian N., Cortez C., Gaw S.L., Blauvelt C.A. (2026). Regional Variation in Maternal RSV Vaccine Access and Attitudes across Two California Cohorts. Prev. Med. Rep..

[91] Bizot E., Portet Sulla V., Vauloup-Fellous C., Rameix-Welti M., Gajdos V., Seddiki N. (2025). Prophylactic Monoclonal Antibodies against Respiratory Syncytial Virus in Early Life: An In-depth Review of Mechanisms of Action, Failure Factors, and Future Perspectives. Pediatr. Allergy Immunol..

[92] Jabagi M.-J., Bertrand M., Gabet A., Kolla E., Olié V., Zureik M. (2025). Nirsevimab vs. RSVpreF Vaccine for Respiratory Syncytial Virus–Related Hospitalization in Newborns. JAMA.

[93] Moline H.L., Tannis A., Goldstein L., Englund J.A., Staat M.A., Boom J.A., Selvarangan R., Michaels M.G., Weinberg G.A., Halasa N.B. (2025). Effectiveness and Impact of Maternal RSV Immunization and Nirsevimab on Medically Attended RSV in US Children. JAMA Pediatr..

[94] Calomfirescu-Avramescu A., Toma A.I., Mehedințu C., Năstase L., Dima V. (2025). Adherence to Palivizumab for Respiratory Syncytial Virus Prophylaxis in Romanian Infants. Vaccines.

[95] Feitosa D.C., Vieira S.E. (2025). Challenges in the Prophylaxis of Severe Respiratory Syncytial Virus Infections. J. Pediatr..

[96] Ocana de Sentuary C., Testard C., Lagrée M., Leroy M., Gasnier L., Enes-Dias A., Leruste C., Diallo D., Génin M., Rakza T. (2025). Acceptance and Safety of the RSV-Preventive Treatment of Newborns with Nirsevimab in the Maternity Department: A Prospective Longitudinal Cohort Study in France. EClinicalMedicine.

[97] Fitzpatrick T., Parsons Leigh J., Brundin-Mather R., MacDonald J., Blanchard W., Bolotin S., Buchan S.A., Brousseau N., Castillo E., Comeau J.L. (2026). Parental Acceptability of New RSV Preventive Therapies for Infants: A Cross-Sectional Survey in Canada. Hum. Vaccin. Immunother..

[98] Ortiz-Prado E., Suárez-Sangucho I.A., Vasconez-Gonzalez J., Santillan-Roldán P.A., Villavicencio-Gomezjurado M., Salazar-Santoliva C., Tello-De-la-Torre A., Izquierdo-Condoy J.S. (2025). Pandemic Paradox: How the COVID-19 Crisis Transformed Vaccine Hesitancy into a Two-Edged Sword. Hum. Vaccin. Immunother..

[99] European Centre for Disease Prevention and Control Measles on the Rise Again in Europe: Time to Check Your Vaccination Status. https://www.ecdc.europa.eu/en/news-events/measles-rise-again-europe-time-check-your-vaccination-status.

[100] Davitoiu A.-M., Spatariu L., Plesca D.-A., Dimitriu M., Cirstoveanu C., Chindris S. (2021). Review of the Measles Epidemic in Children from Central Eastern Europe in the Third Millennium. Exp. Ther. Med..

[101] Rosca I., Turenschi A., Dinulescu A., Lichii V. (2025). The Re-Emergence of Pediatric Pertussis: Insights from a Regional Romanian Hospital. Antibiotics.

[102] Holt E. (2024). Pertussis Outbreak in Czech Republic. Lancet Infect. Dis..

[103] European Centre for Disease Prevention and Control Increase of Pertussis Cases in the EU/EEA. https://www.ecdc.europa.eu/en/publications-data/increase-pertussis-cases-eueea.

[104] Poeta M., Moracas C., Albano C., Petrarca L., Maglione M., Pierri L., Carta M., Montaldo P., Venturini E., De Luca M. (2024). Pertussis Outbreak in Neonates and Young Infants across Italy, January to May 2024: Implications for Vaccination Strategies. Eurosurveillance.

[105] Ferreira Caceres M.M., Sosa J.P., Lawrence J.A., Sestacovschi C., Tidd-Johnson A., Rasool M.H.U., Gadamidi V.K., Ozair S., Pandav K., Cuevas-Lou C. (2022). The Impact of Misinformation on the COVID-19 Pandemic. AIMS Public Health.

[106] Gates D.M., Cohen S.A., Orr K., Caffrey A.R. (2022). Pediatric Influenza Vaccination Rates Lower than Previous Estimates in the United States. Vaccine.

[107] Jugulete G., Olariu M.C., Stanescu R., Luminos M.L., Pacurar D., Pavelescu C., Merișescu M.-M. (2024). The Clinical Effectiveness and Tolerability of Oseltamivir in Unvaccinated Pediatric Influenza Patients during Two Influenza Seasons after the COVID-19 Pandemic: The Impact of Comorbidities on Hospitalization for Influenza in Children. Viruses.

[108] Burgaya-Subirana S., Balaguer M., Miró Catalina Q., Sola L., Ruiz-Comellas A. (2024). Influenza Vaccination Coverage in Children: How Has COVID-19 Influenced It? A Review of Five Seasons (2018–2023) in Central Catalonia, Spain. Vaccines.

[109] Moulia D.L., Link-Gelles R., Chu H.Y., Jamieson D., Brooks O., Meyer S., Weintraub E.S., Shay D.K., Prill M.M., Thomas E.S. (2025). Use of clesrovimab for prevention of severe respiratory syncytial virus–associated lower respiratory tract infections in infants: Recommendations of the Advisory Committee on Immunization Practices—United States, 2025. MMWR Morb. Mortal. Wkly. Rep..

[110] Madhi S.A., Simões E.A.F., Acevedo A., Novoa Pizarro J.M., Shepard J.S., Railkar R.A., Cao X., Maas B.M., Zang X., Krick A. (2025). A phase 1b/2a trial of a half-life–extended respiratory syncytial virus neutralizing antibody, clesrovimab, in healthy preterm and full-term infants. J. Infect. Dis..

[111] Zar H.J., Simões E.A.F., Madhi S.A., Ramilo O., Senders S.D., Shepard J.S., Laoprasopwattana K., Piedrahita J., Novoa J.M., Vargas S.L. (2025). Clesrovimab for prevention of respiratory syncytial virus disease in healthy infants. N. Engl. J. Med..

